# Socioecological influencers of health-promoting lifestyles in Chinese: a preliminary survey using convenient samples

**DOI:** 10.3389/fpubh.2023.1309824

**Published:** 2024-01-08

**Authors:** Li Huang, Hansen Li, Haowei Liu, Haodong Tian, Haoyue Luo, Jinlong Wu, Yue Luo, Li Peng, Liya Guo

**Affiliations:** ^1^College of Physical Education, Southwest University, Chongqing, China; ^2^Key Lab of Physical Fitness Evaluation and Motor Function Monitoring, Southwest University, Chongqing, China; ^3^Chongqing College of International Business and Economics, Chongqing, China; ^4^College of Physical Education, Yili Normal University, Xinjiang, China

**Keywords:** health-promoting lifestyles, health behavior, ecological model of health behavior, a crosssectional study, health-related behaviors

## Abstract

**Background:**

Healthy lifestyles are considered important means to reduce the burden of diseases. This cross-sectional study was conducted based on the Ecological Model of Health Behavior (EMHB) to analyze the factors associated with the health-promoting lifestyles of Chinese residents.

**Methods:**

We carried out a cross-sectional investigation in July 2023. Our investigated factors included social-demographic characteristics (including sex, age, education level, employment status, marital status, personal monthly income, and daily behavioral habits [which were measured by a questionnaire)], health literacy [which was measured by the Chinese version of the Health Literacy Scale Short-Form scale (HLS-SF12)], and family health [which was measured by the Chinese version of the Short-Form of the Family Health Scale (FHS-SF)]. Our outcome was health promoting lifestyle, which was measured by a revised version of Health Promoting Lifestyle Profile-II (HPLP-IIR). Data were analyzed using stepwise regression.

**Results:**

A total of 1,402 participants were enrolled. Higher scores of HLS-SF12 (*β* = 0.467), having regular exercise (*β* = 0.212), and regular physical examination (*β* = 0.088) were associated with better health-prompting lifestyles. However, older age (≥60 years) (*β* = −0.046), drinking (*β* = −0.066), and sleeping time (5–6 h/day) (*β* = −0.048) were associated lower levels of health-prompting lifestyles. Living with family (*β* = 0.077), FHS-SF (*β* = 0.104), and married (*β* = −0.077) were significant influencers. Unemployed (*β* = −0.048), receiving retirement pay (*β* = −0.053), and economic support provided by parents (*β* = 0.094) were associated with better health-prompting lifestyles. There were multiple influencing factors of the six dimensions of the HPLP-IIR. Our findings indicate that community residents with higher health literacy, better family health, and health-related behaviors tend to have better health-promoting lifestyles.

**Conclusion:**

Our findings have confirmed the complex impacts of social-ecological factors on health-promoting lifestyles, which may help policy makers with health-promotion strategies making and also help researchers to control for confounding in study design.

## 1 Introduction

Lifestyle has been defined as all those behaviors over which an individual has control, including actions that affect a person’s health risks, and as discretionary activities with significant impact on health status that are a regular part of one’s daily pattern of living (1). A health-promoting lifestyle is one in which self-initiated, continuous, daily activity is undertaken with the deliberate aim of increasing or promoting an individual’s health and well-being (2). According to the World Health Organization (WHO), approximately 60% of factors related to individual health and quality of life are correlated with lifestyle choices (3). Specifically, studies have suggested that many lifestyle factors, such as not smoking, not using alcohol, and engaging in physical exercise, are contributing to the promotion of overall health, such as lowering the risks of cardiovascular disease, mental disorders, and all-case fatality (4, 5). For these reasons, considerable efforts have been made to explore factors that are likely to enhance health-promoting lifestyles. For instance, Mei et al. (6) found that various demographic variables such as sex, age, personal characteristics, smoking, drinking alcohol, and marital status can influence the eating behavior of adults (6). Likewise, Silvanus et al. (7) found that age, family history of diabetes, non-smoking status, and low family income are potential influencers of regular seeking behavior (7). Moreover, Jusoh et al. (8) found that marital status, parents’ practice, peer practice and education significantly influenced women inmates to smoke (8). However, little attention has been paid to the overall lifestyles that contain multiple dimensions (e.g., interpersonal relationships, nutrition intake, and, physical activity engagement). Moreover, while some studies have examined the associations between sociodemographic factors (e.g., age, sex, and education) and health promoting-lifestyles (e.g., taking a balanced diet, participating in physical activity, and improving interpersonal relationships), other factors that come from a lager range of social and physical background, namely social ecological factors (e.g., family and neighborhood/community variables), are rarely considered in such a research context. Most importantly, the existing evidences are primarily concentrated in European and American countries, whereas the influencing factors in the general Chinese populations have been insufficiently studied. The “Report on Nutrition and Chronic Disease Status of Chinese Residents (2020)” indicates that Chinese residents generally exhibit unhealthy lifestyles, which have contributed to a continuous increase in the prevalence of chronic diseases in China (9). This fact further highlights the necessity of relevant explorations among Chinese populations.

In recent years, the importance of healthy lifestyles in reducing the burden of diseases has gained significant attention. To understand the complex interplay of factors influencing health-promoting lifestyles, the application of ecological models of behavior has been widely recognized. The Ecological Model of Health Behavior (EMHB) provide is a framework that helps understand the complex interactions between individuals and their environment in relation to health behaviors. In China, the EMHB has primarily been utilized to explore factors related to chronic diseases (10–12), comorbidities (13, 14), physical inactivity (15), and quality of life among individuals with chronic conditions (16). However, there is a significant gap in the exploration of social ecological factors influencing lifestyle behaviors. Specifically, limited research has focused on the comprehensive examination of factors associated with health-promoting lifestyles among the general population, encompassing a range of social and physical dimensions. Therefore, this study is presented to address these gaps by conducting a preliminary investigation using the ecological model of behavior to identify the potential socioecological influencers on health-promoting lifestyles among a subset of Chinese residents.

## 2 Theoretical basis

EMHB is widely recognized as an effective framework for identifying the factors that influence health behavior at various levels and establishing connections among individual, social behaviors and environmental determinants (17). There are several variants of the EMHB, but in general, the levels consist of individual, interpersonal, organizational, community, and policy (18).

In relevant studies, the EMHB was usually employed to guide the selection of factors prior to the investigation (6, 19–21). In practice, there are five levels to be considered. The first level is individual characteristics, such as sex, age, body mass index (BMI), education level, and more. The second level contains individual behaviors, such as smoking, drinking, exercise, and more. The third level is interpersonal networks, including marital status, family health, living status, and more. The fourth level is community, which usually contains variables such as occupation and income. The fifth level refers to the policy environment, which can include economic, social, cultural, and policy-related factors at the community, government, national, and even global levels (22).

**Figure 1:**
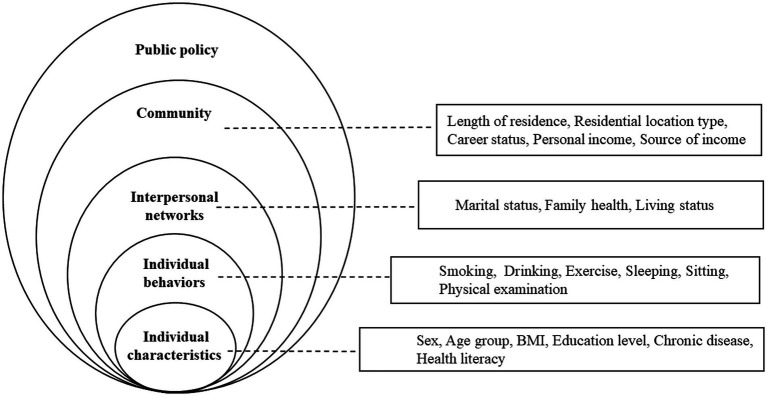
Factors associated with lifestyle based on the ecological model of health behavior [Source: Adapted from Mei et al. (6), with modifications].

In this study, we considered four levels (Figure 1), which were: individual characteristics, individual behaviors, interpersonal networks, and community levels. The first level, namely individual characteristics, included sex, age, BMI, education level, chronic condition, and health literacy. Most of these factors have been demonstrated to be associated with health-promoting lifestyles. It is noteworthy that, health literacy, which usually refers to cognitive and social skills determining individuals’ motivation and ability to access, understand, and use the information to maintain and promote their health, has been rarely studied in Chinese communities (23). In the second and individual behaviors level, we considered smoking, drinking, regular exercise, sleep duration, sitting/sedentary duration, and regular physical examination. The third level (interpersonal networks level) included marital status, family health, and living status (e.g., living alone or not). These factors may reflect family members’ interactions as well as their emotional and economic status. In this way, they may indicate family health (24). In the fourth level (community level), the length of residence, residential locations (e.g., rural or urban), career status, personal income, and source of income were considered. These factors are common community-level determinants that can affect individuals’ health and behaviors.

Our study was conducted with the aim of exploring the association between these socioecological factors and health-promoting behaviors among Chinese residents, based on the theoretical framework described above.

## 3 Materials and methods

### 3.1 Study design

Our survey was conducted from July to September 2023. We used online convenience sampling for our survey. During the recruitment, we collaborated with faculty members from several universities in China to recruit participants among their communities and used a snowball strategy to attract more participants through the social circles of the initially recruited participants. Convenience sampling was chosen for its practicality and ease of implementation, allowing us to efficiently gather data and complete the survey. Our study utilized the popular survey software in China^1^. The questionnaire was distributed based on the seven major geographical regions of China: Northeast China, North, Central, South, East, Northwest, and Southwest. To ensure data integrity, we monitored participants’ devices and IP addresses, allowing each user to complete the survey only once. All collected data were treated with strict confidentiality measures. Prior to providing genuine responses, participants were required to sign an informed consent form to proceed with the survey. We strictly maintained the confidentiality of all data.

### 3.2 Participants

Our inclusion criteria were: (1) Age 18 and above (the legally defined adults in Chinese law), (2) Voluntary participation in this study and confirmation of informed consent, and (3) Residing in communities (defined by the Chinese government) in China. Our exclusion criteria were (1) completion times of less than 270 s (this is the basic time needed for completing the questionnaire according to our pre-testing) and (2) questionnaires displaying patterns of consistent or automated responses (monitored by the questionnaire platform). We initially included residents from 290 cities in 21 provinces, 5 autonomous regions, and 4 municipalities, resulting in a total of 1,538 community residents. After removing those subjects based on our exclusion criteria, a total of 1,402 residents were included in the final analysis.

### 3.3 Variables and measurement

#### 3.3.1 Predictor/independent variables

(1) Individual characteristics: sex, age group, BMI, education level, whether having been diagnosed chronic condition, and health literacy were self-reported in the survey. Personal age group was categorized into five types (1 = 18–29 years, 2 = 30–39 years; 3 = 40–49 years;4 = 50–59 years;5 ≥ 60 years). BMI is calculated by taking a person’s weight, in kilograms, divided by their height, BMI = weight (in kg)/ height^2 (in m^2). According to Chinese adult standards, BMI was grouped as underweight (BMI of <18.5), normal weight (18.5 ≤ BMI < 24), overweight (24 ≤ BMI ≤ 28), and obesity (BMI > 28) (6). Education level was self-reported and categorized into six types (1 = elementary school or lower, 2 = junior high school; 3 = high school /technical secondary school; 4 = junior college; 5 = undergraduate; 6 = postgraduate). Health literacy was measured by the Chinese version of HLS-SF12 translated by Sun et al. (24). The scale consists of three dimensions: health care, disease prevention, and health promotion, comprising a total of 12 items. Each item is scored on a 4-point scale, with response options ranging from 1 (very difficult) to 4 (very easy), using a formula to calculate a standardized health literacy index ranging from 0 to 50, with a higher scores representing higher level of health literacy. The formula is, index = (mean − 1) * (50/3), where the mean is the average of all items involved for each individual (25). The HLS-SF12 was developed by Duong et al., which is an abbreviated and refined measurement tool designed to assess health literacy in a fast, comprehensive, and effective manner (25). The Chinese version of HLS-SF12 serves as a valuable tool for assessing the current status of health literacy in China and identifying influential factors. In this study, the questionnaire showed acceptable internal consistency (Cronbach’s *α* = 0.931).

(2) Individual behaviors: smoking or not (1 = yes, 2 = no), drinking or not (1 = yes, 2 = no), taking regular exercise or not (1 = yes, 2 = no), and taking physical examination regularly or not (1 = yes, 2 = no) were captured by binary choices. Sleeping duration was categorized into four types (1 = less than 5 h/day; 2 = 5–6 h/day; 3 = 6–7 h/day; 4 = more than 7 h/day). Siting duration was categorized into four types (1 = less than 4 h/day; 2 = 4–6 h/day; 3 = 6–8 h/day; 4 = more than 8 h/day).

(3) Interpersonal networks: Marital status was captured by a categorical response scale (1 = unmarried; 2 = married; 3 = divorced; 4 = widowed); living status was categorized into three types (1 = living alone; 2 = living with family; 3 = living in work/school dormitories). Family health was measured using the Short-Form of the Family Health Scale in the Chinese Version (FHS-SF). The Chinese version of FHS-SF was cross-culturally validated by Wang et al. (26). It comprises four dimensions, encompassing a total of 10 items. These dimensions are: (1) Family/Social/Emotional Health Processes (item 1, 2, and 5), (2) Family Health Lifestyle (item 3 and 4), (3) Family Health Resources (item 6, 9, and 10), and (4) External Social Support for the Family (item 7 and 8). The items are rated on a 5-point Likert scale, ranging from “strongly disagree” to “strongly agree.” Notably, items 6, 9, and 10 employ reverse scoring, with higher scores indicating a better family health status (6). The Chinese version of FHS-SF demonstrates good reliability and validity, making it suitable for assessing the level of family health among Chinese residents. In this study, the scale showed acceptable internal consistency (Cronbach’s *α* = 0.737).

(4) Community: the length of residence was categorized into five types (1 = 0–5 years; 2 = 6–10 years; 3 = 11–15 years; 4 = 16–20 years; 5 = More than 20 years). Residential location was categorized into two types (1 = urban; 2 = rural). Career status was categorized into two types (1 = student; 2 = full time; 3 = part time; 4 = unemployed; 5 = retired; 6 = famer). Monthly personal income was categorized into five types (1 = ≤3,000 RMB; 2 = 3,001–5,000 RMB;3 = 5,001 ~ 8,000 RMB; 4 = 8,001 ~ 12,000 RMB; 5 = >12,000 RMB). According to the data from the National Bureau of Statistics, the wage *income per capita* of residents in China in the first three quarters was 16,747 RMB (Monthly income ≈ 16,747 RMB / 3 = 5,582.33 RMB). One RMB is approximately 0.14 USD or 0.12 EUR (September 2023). Source of income was divided into six categories: 1 = salary; 2 = parental support; 3 = pension/retirement benefits; 4 = support from children or relatives; 5 = government subsidies; 6 = other sources of income.

#### 3.3.2 Outcome/dependent variables

The revised version of Health Promoting Lifestyle Profile-II (HPLP-II R) was used for the assessment of health-promoting lifestyles. This questionnaire is a well-validated instrument that evaluates individuals’ health-promoting behaviors across multiple dimensions (27). It is an adapted version of the 52-item HPLP-II, specifically tailored for the Chinese population by Cao et al. (28). This questionnaire consists of six dimensions: Interpersonal Relationships (5 items), Nutrition (6 items), Health Responsibility (11 items), Physical Activity (8 items), Stress Management (5 items), and Self-actualization (5 items). Each item is rated on a 4-point Likert-type scale, where 1 indicates “never” and 4 indicates “always.” The total score ranges from 40 to 160. Scores ranging from 40 to 80, 81–120, and 121–160 correspond to low, moderate, and high levels of health-promoting lifestyle, respectively (29). In this study, the scale showed excellent internal consistency (Cronbach’s *α* = 0.96). Meanwhile, the sub-scales for the six dimensions, including Interpersonal Relationships (Cronbach’s *α* = 0.822), Stress Management (Cronbach’s *α* = 0.791), Health Responsibility (Cronbach’s *α* = 0.902), Nutrition (Cronbach’s *α* = 0.788), Physical Activity (Cronbach’s *α* = 0.888), and Self-actualization (Cronbach’s *α* = 0.850) also showed acceptable internal consistency.

### 3.4 Quality control

Several measures have been implemented for quality control. Firstly, a pilot survey was conducted to validate and refine the questionnaire. Feedback was gathered from a random sample of 30 participants, assessing the clarity and relevance of the survey content. Necessary adjustments and improvements were made based on their feedback. Secondly, as demonstrated in our exclusion criteria, a time limit was established through a pre-test involving multiple participants from different age groups. Surveys completed within 270 s were excluded as this timeframe was deemed inadequate for thoughtful and considered responses. This time restriction aims to prevent hasty answers that might increase common method bias.

### 3.5 Statistical methods

Descriptive statistics were utilized to calculate the number and percentage of categorical variables, while continuous variables were represented by means and standard deviations. Multiple linear regression was selected for analysis, which is particularly useful for identifying the associations between variables. Linear regression exhibits considerable robustness to non-normal data, especially when the sample size for parameter estimation is relatively large (where the number of observations per variable is > 10) (30). Therefore, we did not transform the data before testing.

Prior to the regression, we tested multicollinearity according to the variance inflation factor (VIF), where a VIF value smaller than 5.0 was considered as the absence of multicollinearity issue (31). Based on this rule, we found no risk of multicollinearity (our VIFs were smaller than 2.0). Unordered categorical variables were processed into dummy variables before the analysis.

Based on the method of others (32, 33), we adopted a forward stepwise regression approach. In this approach, the model starts with no independent variables, and at each step, the variable that provides the best improvement in the model’s fit (e.g., reduces the residual sum of squares) is added. This process continues until adding more variables no longer significantly improves the model.

### 3.6 Ethics statement

This study obtained approval from the Ethics Committee of Southwest University Hospital, Chongqing, China (SWU-ETF-2023-07-17-011). The methods involved in our research were conducted in accordance with the guidelines and regulations outlined in the Helsinki Declaration.

## 4 Results

### 4.1 Participants’ characteristics

Table 1 presents the characteristics of the participants. A total of 1,402 individuals were included in our study. 64.1% of participants were male, 89.3% were undergraduate or higher education level, and 85.3% lived in urban areas. The mean score for the participants’ HLS-SF12 was 37.9, FHS-SF was 37.53, and the HPLP-IIR was 111.19. Interpersonal relations showed the highest score, and health responsibility showed the lowest score, with physical activity ranking the second lowest.

**Table 1:** Participants’ characteristics.

Categorical variables	Category	*N*	Percentage
**Sex**	Male	898	64.1%
	Female	504	35.9%
**BMI**			
	Underweight (<18.5)	75	5.3%
	Normal weight (18.5–24)	799	57.0%
	Overweight (24–28)	425	30.3%
	Obese (≥28)	103	7.3%
**Education level**			
	≤Elementary school	8	0.6%
	Junior high school	24	1.7%
	High school/technical secondary school	39	2.8%
	Junior college	80	5.7%
	Undergraduate	789	56.3%
	Postgraduate	462	33.0%
**Age group (year)**			
	18–29	621	44.3%
	30–39	273	19.5%
	40–49	247	17.6%
	50–59	172	12.3%
	≥60	89	6.3%
**Marital status**			
	Unmarried	567	40.4%
	Married	788	56.2%
	Divorced	37	2.6%
	Widowed	10	0.7%
**Type of residence**			
	Urban	1,196	85.3%
	Rural	206	14.7%
**Living situation**			
	Living alone	147	10.5%
	Live with family	1,123	80.1%
	Living in work/school dormitories	132	9.4%
**Length of residence**			
	0–5 years	256	18.3%
	6–10 years	217	15.5%
	11–15 years	164	11.7%
	16–20 years	240	17.1%
	More than 20 years	525	37.4%
**Working status**			
	Student	330	23.5%
	Full time	897	64.0%
	Part time	23	1.6%
	Unemployed	40	2.9%
	Retired	99	7.1%
	Famer	13	0.9%
**Monthly personal income (RMB)**			
	≤3,000	357	25.5%
	3,001–5,000	195	13.9%
	5,001–8,000	332	23.7%
	8,001–12,000	285	20.3%
	>12,000	233	16.6%
**Economic sources**	Salary	949	67.7%
	Parental support	313	22.3%
	Pension/Retirement benefits	76	5.4%
	Support from children or relatives	3	0.2%
	Government subsidies	11	0.8%
	Other sources of income	50	3.6%
**Smoking**			
	Yes	347	24.8%
	No	1,055	75.2%
**Drinking**			
	Yes	612	43.7%
	No	790	56.3%
**Exercise regularly**			
	Yes	777	55.4%
	No	625	44.6%
**Regular physical examination**			
	Yes	822	58.6%
	No	580	41.4%
**Sitting time**			
	Less than 4 h/day	378	27.0%
	4–6 h/day	576	41.1%
	6–8 h/day	304	21.7%
	More than 8 h/day	144	10.3%
**Sleeping time**			
	Less than 5 h/day	14	1.0%
	5–6 h/day	173	12.3%
	6–7 h/day	593	42.3%
	More than 7 h/day	622	44.4%
**Chronic condition**			
	Yes	216	15.4%
	No	1,186	84.6%
**Continuous variables**	***M* (SD)**	**Median(IQR)**	**Range**
HLS-SF12(index)	36.02 (7.92)	33.33 (8.33)	0–50
FHS-SF(score)	37.53 (6.35)	37(8)	20–50
**Dimensions**	***M* (SD)**	**SS score**	**Rank**
Interpersonal relations	15.28 (2.55)	76.42	1
Health responsibility	27.79 (6.19)	63.16	6
Stress management	14.17 (2.67)	70.83	4
Nutrition	18.22 (2.94)	75.92	2
Physical activity	20.80 (5.00)	65.00	5
Self-actualization	14.93 (2.80)	74.65	3
HPLP**-**II R total score	111.19 (18.84)	69.50	–

### 4.2 The factors relevant to the HPLP-II R scores

Based on the EMHB, our study found 12 significant factors of the health-promoting lifestyles (Table 2). In the first level, People of older age (≥60 years) (*β* = −0.046) tended to have an unhealthy lifestyle. Participants who had higher scores of HLS-SF12 (*β* = 0.467) were more likely to have better lifestyles. In the second level, participants who exercised regularly (*β* = 0.212) and had physical examination regularly (*β* = 0.088) tended to have better lifestyles. Participants with shorter sleeping time (5–6 h/day) (*β* = −0.048), or drinking (*β* = −0.066) tended to have worse lifestyles. In the third level, participants who lived with family (*β* = 0.077), and had higher scores of FHS-SF (*β* = 0.104) had better lifestyles. Participants who were married (*β* = −0.077) showed lower HPLP-II R scores. At the fourth level, participants who were unemployed (*β* = −0.048), and receiving retirement pay (*β* = −0.053) showed lower HPLP-II R scores. However, the participants who had higher economic support provided by parents (*β* = 0.094) showed higher scores of the HPLP-II R.

**Table 2:** The stepwise regression analysis of factors associated with HPLP-II R total score.

Variables	Coef.	*β*	*t*	*p*
Individual characteristics				
Age (Ref: 18–29, year)				
≥60	−2.658	−0.046	−2.116	0.035
HLS-SF12	1.112	0.467	20.812	<0.001
Individual behaviors				
Whether exercise regularly (Ref: No)				
Yes	8.040	0.212	9.726	<0.001
Whether regular physical examination (Ref: No)				
Yes	3.350	0.088	3.665	<0.001
Whether drinking(Ref: No)				
Yes	−2.495	−0.066	−3.117	0.002
Sleeping time (Ref: Less than 5 h/day)				
5–6 h/day	−2.761	−0.048	−2.295	0.022
Interpersonal networks				
Marital status (Ref: Unmarried)				
Married	−2.919	−0.077	−2.448	0.014
Living situation (Ref: Living alone)				
Living with family	3.618	0.077	3.133	0.002
FHS-SF	0.307	0.104	4.454	<0.001
Community				
Career status (Ref: Student)				
Unemployed	−5.455	−0.048	−2.309	0.021
Economic sources (Ref: Salary)				
Provided by parents	4.266	0.094	3.387	0.001
Retirement pay	−4.429	−0.053	−2.505	0.012

### 4.3 The factors relevant to the dimensions of HPLP-II R scores

Based on the EMHB, our study found factors of the dimensions of health-promoting lifestyles (Table 3). In the interpersonal relationships, we found 10 significant factors. In the first level, being female (*β* = 0.050) and had higher scores of HLS-SF12 (*β* = 0.459) were associctated better lifestyles. Participants who had older age (≥60 years) (*β* = −0.061) and had a junior high school educational level (*β* = −0.051) tended to have an unhealthy lifestyle. In the second level, Participants who exercised regularly (*β* = 0.069) had better interpersonal relationships. In the third level, participants who had higher scores of FHS-SF (*β* = 0.242) tended to have better interpersonal relationships. At the fourth level, participants who were unemployed (*β* = −0.043), relied on retirement income (*β* = −0.071), and had a monthly income of 5,001 ~ 8,000 RMB (*β* = −0.053) showed lower scores in interpersonal relationships. Participants who received economic support from their parents (*β* = 0.094) showed higher scores on the interpersonal relationships.

**Table 3:** The factors relevant to the dimensions of HPLP -II R scores.

Variables	Coef.	*β*	*t*	*p*
**Interpersonal relationships**				
Individual characteristics				
Sex (Ref: Male)				
Female	0.265	0.050	2.369	0.018
Age (Ref: 18-29, year)				
≥60	−0.472	−0.061	−2.799	0.005
Education level (Ref: ≤Elementary school)				
Junior high school	−1.007	−0.051	−2.381	0.017
HLS-SF12	0.148	0.459	20.139	<0.001
Individual behaviors				
Whether exercise regularly (Ref: No)				
Yes	0.356	0.069	3.183	0.001
Interpersonal networks				
FHS-SF	0.097	0.242	10.247	<0.001
Community				
Career status (Ref: Student)				
Unemployed	−0.656	−0.043	−2.001	0.046
Economic sources (Ref: Salary)				
Provided by parents	0.576	0.094	4.018	<0.001
Retirement pay	−0. 801	−0.071	−3.317	0.001
monthly income (Ref:≤3000yuan)				
5,001 ~ 8000yuan	−0. 316	−0.053	−2.381	0.017
**Health responsibility**	**Coef.**	**β**	**t**	***p***
Individual characteristics				
Age (Ref: 18–29, year)				
≥60	−1.133	−0. 060	−2.510	0.012
Education level (Ref: ≤Elementary school)				
Junior high school	−2.344	−0.049	−2.134	0.033
HLS-SF12	0.300	0.384	15.404	<0.001
Individual behaviors				
Whether exercise regularly (Ref: No)				
Yes	2.020	0.162	6.687	<0.001
Whether regular physical examination (Ref: No)				
Yes	1.836	0. 146	5.666	<0.001
Whether drinking(Ref: No)				
Yes	−0.981	−0.079	−3.083	0.002
Whether smoking (Ref: No)				
Yes	−0.721	−0.050	−1.994	0.046
Sitting time (Ref: Less than 5 h/day)				
More than 8 h/day	−1.286	−0.063	−2.729	0.006
Sleeping time (Ref: Less than 5 h/day)				
5–6 h/day	−0.877	−0.047	−1.999	0.046
Interpersonal networks				
Marital status (Ref: Unmarried)				
Widowed	−3.883	−0.053	−2.292	0.022
FHS-SF	−0.054	−0.056	−2.167	0.030
Community				
Economic sources (Ref: Salary)				
Provided by parents	1.445	0.097	3.747	<0.001
Retirement pay	−2.329	−0.085	−3.625	<0.001
**Stress Management**	**Coef.**	**β**	**t**	***p***
Individual characteristics				
Education level (Ref: ≤ Elementary school)				
Junior college	−0.651	−0.057	−2.572	0.010
BMI (Ref: Normal weight (18.5–24))				
Underweight (<18.5)	−0.536	−0.045	−1.999	0.046
HLS-SF12	0.142	0.421	17. 557	<0.001
Individual behaviors				
Whether exercise regularly(Ref: No)				
Yes	0. 706	0.132	5.781	<0.001
Sleeping time (Ref: Less than 5 h/day)				
5 ~ 6 h/day	−0. 441	−0.054	−2.446	0.015
Interpersonal networks				
Marital status (Ref: Unmarried)				
Married	−0. 657	−0.122	−3.724	<0.001
Living situation (Ref: Living alone)				
Live with family	0.517	0. 077	2.958	0.003
FHS-SF	0.053	0.127	5.120	<0.001
Community				
Economic sources (Ref: Salary)				
Provided by parents	0.746	0.116	3.914	<0.001
Monthly income (Ref: ≤3000 yuan)				
>12,000 yuan	−0.357	−0.050	−2.169	0.030
**Nutrition**	**Coef.**	**β**	**t**	***p***
Individual characteristics				
Sex (Ref: Male)				
Female	0.411	0.067	2.685	0.007
BMI (Ref: Normal weight (18.5–24))				
Overweight (24 ~ 28)	0.317	0.050	2.170	0.030
HLS-SF12	0.153	0.412	17.748	<0.001
Individual behaviors				
Whether exercise regularly (Ref: No)				
Yes	0.438	0.074	3.286	<0.001
Whether regular physical examination (Ref: No)				
Yes	0.330	0.055	2.279	0.023
Whether drinking (Ref: No)				
Yes	−0.516	−0.087	−3.466	0.001
Whether smoking (Ref: No)				
Yes	−0.520	−0.076	−3.144	0.002
Sleeping time (Ref: Less than 5 h/day)				
5–6 h/day	−0.441	−0.049	−2.283	0.023
Interpersonal networks				
Living situation (Ref: Living alone)				
Live with family	0. 511	0.069	3.181	0.001
FHS-SF	0.104	0.224	9.407	<0.001
Community				
Economic sources (Ref: Salary)				
Provided by parents	0.455	0.064	2.671	0.008
**Physical activity**	**Coef.**	**β**	**t**	***p***
Individual characteristics				
Sex (Ref: Male)				
Female	−0.639	−0.061	−2.646	0.008
Education level (Ref: ≤Elementary school)				
Junior high school	−2.005	−0. 052	−2.403	0. 016
Whether diagnosed Chronic condition (Ref: No)				
Yes	−0. 850	−0.061	−2.816	0. 005
HLS-SF12	0. 213	0. 338	15.293	<0.001
Individual behaviors				
Whether exercise regularly (Ref: No)				
Yes	3.436	0. 342	14.988	<0.001
Whether regular physical examination (Ref: No)				
Yes	0. 839	0.083	3.442	0. 001
Whether smoking (Ref: No)				
Yes	0.549	−0.047	2.040	0.042
Sitting time (Ref: Less than 5 h/day)				
6–8 h/day	−0.895	−0.071	−3.241	0.001
More than 8 h/day	−1.324	−0.080	−3.625	<0.001
Community				
Economic sources (Ref: Salary)				
Provided by parents	1.614	0.134	5.736	<0.001
**Self-actualization**	**Coef.**	***β***	***t***	***p***
Individual characteristics				
Sex (Ref: Male)				
Female	0.274	0.047	2.189	0.029
Education level (Ref: ≤Elementary school)				
Junior college	−0.573	−0.047	−2.196	0.028
Whether diagnosed Chronic condition (Ref: No)				
Yes	−0.333	−0.043	−1.982	0.048
HLS-SF12	0.149	0.421	18.238	<0.001
Individual behaviors				
Whether exercise regularly (Ref: No)				
Yes	0.818	0.145	6.613	<0.001
Interpersonal networks				
FHS-SF	0.104	0.236	9.939	<0.001
Community				
Economic sources (Ref: Salary)				
Provided by parents	0.693	0.103	4.553	<0.001

In the dimension of health responsibility, we found 13 factors. In the first level, participants who were older (≥60 years) (*β* = −0.060) and had a junior high school education level (*β* = −0.049) tended to have worse health responsibility. Participants who had higher scores of HLS-SF12 (*β* = 0.384) showed better health responsibilities. In the second level. Participants who exercised regularly (*β* = 0.162) and had physical examination regularly (*β* = 0.146) showed better health responsibility. However, participants who had been smoking (*β* = −0.050), drinking (*β* = −0.079), sitting for more than 8 h (*β* = −0.063), and sleeping for 5–6 h (*β* = −0.047) had worse health responsibility. In the third level, the participants who were widowed (*β* = −0.053) and had lower scores of FHS-SF (*β* = −0.056) tended to have worse health responsibilities. At the fourth level, participants who received economic support from their parents (*β* = 0.097) showed higher scores on health responsibility. Conversely, the participants who relied on retirement income (*β* = −0.085) showed worse health responsibility.

In the dimension of stress management, we found 10 factors. In the first level, the participants who had higher scores of HLS-SF12 (*β* = 0.421) tended to have better stress management. However, those who were underweight (<18.5) (*β* = −0.045) and had a junior college education level (*β* = −0.057) showed lower scores on stress management. In the second level, participants who exercised regularly (*β* = 0.132) had better stress management. Conversely, sleeping 5 ~ 6 h (*β* = −0.054) was negatively associated with stress management. In the third level, participants who were married (*β* = −0.122) showed lower scores of stress management. However, Living with family (*β* = 0.077) and having higher scores of FHS-SF (*β* = 0.127) tended to have better stress management. At the fourth level, participants who received economic support from their parents (*β* = 0.116) showed higher scores of stress management. Conversely, participants with monthly income above 12,000 yuan (*β* = −0.050) showed lower scores on stress management.

In the dimension of nutrition, we found 11 factors. In the first level, participants who be female (*β* = 0.067) and were overweight (24–28) (*β* = 0.050), and had higher scores of HLS-SF12 (*β* = 0.412) showed higher scores of nutrition. In the second level, participants who exercised regularly (*β* = 0.086), and had physical examination regularly (*β* = 0.055) showed higher scores on the nutrition, while who had been smoking (*β* = −0.076), drinking (*β* = −0.087), and sleeping 5–6 h (*β* = −0.049) showed lower scores on nutrition. In the third level, participants who lived with family (*β* = 0.069) and had higher scores of FHS-SF (*β* = 0.224) had better nutrition. At the fourth level, participants who received economic support from their parents (*β* = 0.064) showed higher scores on nutrition.

In the dimension of physical activity, we found 10 factors. In the first level, participants who had higher scores of HLS-SF12 (*β* = 0.338) showed higher scores on physical activity. Moreover, females (*β* = −0.061), individuals diagnosed with chronic condition (*β* = −0.061) and those with a junior college education level (*β* = −0.052) exhibited lower scores of physical activity. In the second level, participants who exercised regularly (*β* = 0.342) and had physical examination regularly (*β* = 0.083) tended to have better physical activity. Conversely, those who had been smoking (*β* = −0.047), sitting about 6–8 h/day (*β* = −0.071), and more than 8 h/day (*β* = −0.080) showed lower scores physical activity. At the fourth level, participants who received economic support from their parents (*β* = 0.134) showed higher scores on physical activity.

In the dimension of self-actualization, we found 7 factors. Females (*β* = 0.047) and individuals with higher scores of HLS-SF12 (*β* = 0.421) showed higher scores of self-actualization. The participants who were diagnosed with chronic condition (*β* = −0.043) and those had a junior college educational level (*β* = −0.047) showed lower scores of self-actualization. In the second, third, and fourth levels, just one influencing factor was identified for each. The participants who exercised regularly (*β* = 0.145), had higher scores of FHS-SF (*β* = 0.236), and had economic sources of support from their parents (*β* = 0.103) showed higher scores of self-actualization.

## 5 Discussion

We found that the health-promoting lifestyles of community-dwelling adults in China was at a moderate level (80–120 point), which is consistent with the results of other research (34, 35), indicating that the health lifestyle of Chinese adults is unsatisfactory. Meanwhile, in our study, interpersonal relationships received the highest score, followed by nutrition. In contrast, health responsibility received the lowest score, with physical activity ranking the second lowest. Our findings align with the research by Zhang et al. (36), where interpersonal relationships also received the highest score while health responsibility received the lowest score. Generally, our findings on health-related lifestyle reinforce some previous studies and underline some commonalities among Chinese populations. Based on the EMHB framework, our study explores the factors associated with health-promoting lifestyles across four levels: individual characteristics, individual behaviors, interpersonal networks, and community.

Our research has demonstrated that health-promoting lifestyles were associated with a range of factors. By employing the EMHB model in our study, we identified that these factors are multidimensional and encompass various aspects. It is crucial for future research to consider these holistic perspectives in order to enhance community residents’ lifestyles and devise appropriate intervention strategies. Importantly, it should be noted that different dimensions exhibit slight variations in their influencing factors, underscoring the significance of targeted interventions that account for specific factors pertinent to improving specific health lifestyles among community residents. A potential approach to foster the adoption of healthy lifestyles among community residents is to consider the practice pathway involving the community, family, and individual levels. By implementing interventions and strategies targeting these interconnected levels, there exists the possibility of cultivating positive health behaviors and facilitating the development of sustainable healthy lifestyles within the community.

At the individual characteristic level, sex was found a significant influencing factor of health-promoting lifestyles, with females demonstrating advantages in interpersonal relationships, nutrition, and self-actualization. A study conducted in Japan has revealed that females had higher scores for the six dimensions of HPLP-II and also the total score (37). Consistent findings have also been reported in some other studies on Chinese populations (38, 39). This phenomenon may be attributed to females shouldering more obligations to their families and managing family relationships, which allows them to prioritize dietary choices and interpersonal relationship management (38). Additionally, due to increased independence and autonomy among modern women, they tended to prioritize self-care and self-actualization (39). Education level was also associated with interpersonal relationships, health responsibility, and stress management. This finding may be explained by higher levels of education leading to better acceptance and the ability to effectively access health advice. While research on the relationship between BMI and health-promoting lifestyles is still limited, existing studies have emphasized the importance of addressing obesity and overweight issues in health promotion (40). We found that underweight participants tended to have challenges in effectively managing stress. According to previous studies, underweight and obese individuals showed higher rates of emotional problems compared with normal and overweight participants (41). Additionally, underweight individuals face many other physical health-related risks, such as the higher likelihood of stroke (42), eating slowly (43), and engaging in unhealthy behaviors (44). This may result in individuals with underweight obtaining lower scores in stress management. Age was another contributing factor. Individuals aged 60 and above tended to score lower scores on the HPLP-IIR, interpersonal relationships, and health responsibility compared to their younger counterparts. This finding is consistent with another study (45). Furthermore, patients with chronic diseases exhibited lower scores in the physical exercise and spiritual growth subscales, which is similar to the findings of Aygar’s research (46), where physical activity subscale scores were low among patients with chronic diseases. Additionally, individuals with chronic diseases are more vulnerable to symptoms such as depression and anxiety, which may negatively impact their mental well-being and, consequently, lead to lower scores in the spiritual growth dimension. Furthermore, health literacy is a key factor that impacts the overall score of the HPLP-IIR and its dimensions (47). Individuals with higher health literacy scores tend to have healthier lifestyles. This aligns with previous research that health literacy has demonstrated an association with healthy lifestyles or health-related behaviors (48) In the individual characteristics, we found that factors such as sex, age, education level, BMI, chronic diseases, and health literacy play distinctive roles in the variations of health-promoting lifestyles. Among these factors, health literacy and sex emerge as significant influencers. According to research findings, health literacy is directly associated with disease mortality, overall health status, disease prevention, and health behaviors (49). In future research, it is worth considering various approaches to enhance the health literacy of community residents in order to promote the healthy lifestyles.

At the individual behavioral level, several factors are significantly associated with the health lifestyles of residents, including regular physical examination, smoking, drinking, sleeping, and sedentary behavior. Among them, regular physical exercise showed an impact on the six dimensions of HPLP-IIR and also its total score. Residents who exercised regularly tended to have higher scores, both in total and across each dimension, and those who participated in physical examination regularly showed higher scores of health responsibility, nutrition, and physical activity. Meanwhile, smoking, drinking, sleep patterns, and sedentary behavior were also significantly associated with various dimensions of a health-promoting lifestyle. Numerous studies have demonstrated that adopting healthy behaviors can prolong lifespan (5) and maintain overall health (50). In contrast, unhealthy behaviors, such as smoking, drinking, insufficient exercise, and inadequate sleep are significantly correlated with metabolic syndrome and can therefore impact health (50).

At the interpersonal network level, family health was a crucial factor, which was positively associated with the overall score and the scores in various dimensions of HPLP-IIR. Theoretically, positive family health promotes belonging, caring, and the capacity to perform family responsibilities, which in turn, promotes the health of individual members (51). Furthermore, the effect of participants’ living situation was also significant. Specifically, participants who live with their family tended to have a higher total score, as well as higher scores in nutrition and stress management dimensions compared to those who live alone, live in school/workplace dormitories, or share accommodations with others. This finding is expected because living with family members allows for mutual care and is more conducive to developing healthy lifestyle habits (52). Marital status was also associated with the total score and the scores in health responsibility and stress management dimensions. Specifically, married participants tended to show lower scores in overall and stress management, while widowed participants had lower scores in health responsibility. Similar to our finding, other scholars have also found that widowed individuals scored lower than married or unmarried/divorced individuals across all scales (35).

At the community level, participants who relied on economic support from their parents exhibited higher scores in the total score and six dimensions. Studies have suggested that ideal socioeconomic status was beneficial to healthy behavior (6) and the source of income was a influencing factor of people’s social activity (53). In our study, participants who received financial support from their parents were predominantly college students, and they tended to have a better awareness of health compared to others. This may be a contributing factor to their higher scores in adopting a healthy lifestyle. Additionally, sufficient economic support enables them to fulfill many of their needs in daily life. However, participants relying on retirement pensions tended to have lower scores in interpersonal relationships, health responsibility dimensions, and the total score. This group mainly consists of older adult individuals who had worse health-promoting lifestyles. This finding aligns with several studies conducted in China (34, 45). Moreover, monthly income was associated with interpersonal relationships and stress management. In other words, individuals with higher monthly incomes tended to show worse stress management. This result may come from the fact that higher income levels often coincide with increased work demands and intensity, leading to elevated stress levels and relatively less attention on health-promoting lifestyles (39). Participants with monthly incomes in the range of 5,001–8000 RMB had lower scores in interpersonal relationships, which could result from specific occupational types (54, 55). For example, participants within this salary range may have busy work schedules, more life stress, and fewer financial resources available for social expenses. These may result in weaker interpersonal relationships in their daily lives.

## 6 Limitations

In our study, there are several limitations that warrant consideration. Firstly, due to practical constraints, we were unable to conduct a nationwide random sampling with a large sample size. Consequently, a convenience sample was employed. The gender was not balanced in our sample, which has limited the generalizability of our findings. Caution should therefore be exercised when extrapolating the results to broader contexts. Future research should aim to incorporate more diverse and representative samples to enhance the external validity of the findings.

Secondly, although we used “influencers” as following previous studies to underline the potential roles of these factors in changing residents’ health-promoting behaviors, it should be noted that our findings are solely captured from cross-sectional data with only one observational timepoint, and the nature of such data forbid further causal evaluations. Therefore, all the “effects/influences” we found should be explained as associations. To address this limitation, future investigations could employ longitudinal designs, experimental approaches, or instrumental variables to explore causal relationships and further elucidate the impact of these factors on health behavior.

Additionally, it is noteworthy that we only consider personal or individual factors, while some factors at neighborhood or regional levels, such as regional socioeconomic, have not been included. Since individuals residing in poverty or with low socioeconomic statuses generally exhibit poorer performances in terms of health behavior, these should be taken into consideration in future research.

## 7 Conclusions

This study utilized a cross-sectional design and regression analyses to identify factors likely to influence health-promoting lifestyles. Employing the EMHB model, we identified several factors at individual, interpersonal networks and community levels potentially associated with the six sub-dimensions and overall levels of health-promoting lifestyles among Chinese populations. Our findings may contribute to the development of personalized interventions and controlling confounding effects in research on health-promoting lifestyles. Given our study’s limitations, we advocate for future research with improved samples and methodologies to confirm causal relationships between these factors and health-promoting lifestyles.

## Data availability statement

The original contributions presented in the study are included in the article/supplementary material, further inquiries can be directed to the corresponding author.

## Ethics statement

The studies involving humans were approved by Ethics Committee of Southwest University Hospital. The studies were conducted in accordance with the local legislation and institutional requirements. The participants provided their written informed consent to participate in this study.

## Author contributions

LH: Conceptualization, Data curation, Investigation, Methodology, Software, Writing – original draft, Writing – review & editing. HLi: Writing – review & editing, Formal analysis. HLiu: Investigation, Methodology, Writing – review & editing, Formal Analysis. HT: Formal analysis, Investigation, Methodology, Writing – review & editing. HLu: Formal analysis, Data curation, Investigation, Writing – review & editing. JW: Investigation, Writing – review & editing. YL: Investigation, Writing – review & editing. LP: Funding acquisition, Project administration, Supervision, Writing – review & editing. LG: Data curation, Investigation, Project administration, Supervision, Writing – review & editing.

## Footnotes

^1^ https://www.wjx.cn/
