# Corrigendum: Socioecological influencers of health-promoting lifestyles in Chinese: a preliminary survey using convenient samples

**DOI:** 10.3389/fpubh.2025.1610324

**Published:** 2025-04-25

**Authors:** Li Huang, Hansen Li, Haowei Liu, Haodong Tian, Haoyue Luo, Jinlong Wu, Yue Luo, Li Peng, Liya Guo

**Affiliations:** ^1^College of Physical Education, Southwest University, Chongqing, China; ^2^Key Lab of Physical Fitness Evaluation and Motor Function Monitoring, Southwest University, Chongqing, China; ^3^Chongqing College of International Business and Economics, Chongqing, China; ^4^College of Physical Education, Yili Normal University, Xinjiang, China

**Keywords:** health-promoting lifestyles, health behavior, ecological model of health behavior, a crosssectional study, health-related behaviors

In the published article, there was an error in the Funding statement. A funding reference was incorrect. It originally stated: “Chongqing Municipal Doctoral Research and Innovation Project (No.CTY22100).” The correct Funding statement appears below.

